# Promoting a more diverse and inclusive research workforce through the research scholars program

**DOI:** 10.1186/s12909-024-05075-0

**Published:** 2024-01-30

**Authors:** Nancy E. Schoenberg, Jimmy Robinson, Margaret McGladrey, Lisa A. Cassis, Darwin Conwell, Kevin J. Pearson, Lisa R. Tannock, Donna Wilcock, Stephanie White

**Affiliations:** 1 Department of Behavioral Science, College of Medicine; and Associate Vice President for Research, Center for Health Equity Transformation, Office of the Vice President for Research at the University of Kentucky, Suite 460 Healthy Kentucky Research Building, 760 Press Avenue, Lexington, KY 40536-0679 USA; 2 Department of Sociology, Center for Health Equity Transformation, College of Arts & Sciences at the University of Kentucky, Suite 460 Healthy Kentucky Research Building, 760 Press Avenue, Lexington, KY 40536-0679 USA; 3 Center for Innovation in Population Health and Department of Health Management and Policy, College of Public Health at the University of Kentucky, 111 Washington Ave Suite 205B, Lexington, KY 40508 USA; 4 Department of Pharmacology and Nutritional Science, College of Medicine at the University of Kentucky, 311 Main Building, Lexington, KY 40506-0032 USA; 5 Department of Internal Medicine, College of Medicine at the University of Kentucky, 301 C.T. Wethington Building, 900 Limestone St, Lexington, KY 40536-0200 USA; 6 Department of Pharmacology and Nutritional Science, College of Medicine at the University of Kentucky, 591 C.T. Wethington Building, 900 S. Limestone St, Lexington, KY 40536-0200 USA; 7 Department of Internal Medicine, College of Medicine at the University of Kentucky. Address: C. T, Wethington Building, 900 S. Limestone St, Lexington, KY 40536-0200 USA; 8 Alzheimer’s Disease Research, Department of Neurology at Indianapolis University School of Medicine, GHE 4700, NEUR, Indianapolis, 46202-3082 USA; 9 College of Medicine at the University of Kentucky, 800 Rose St, Lexington, KY 40536 USA

**Keywords:** Diversity, Underrepresentation, Faculty development, Mentoring, Social networking

## Abstract

**Background:**

Novel and comprehensive approaches are needed to address shortcomings in the diversity and inclusiveness of the scientific workforce. In response to this need and informed by multiple programs and data sources, we created the Research Scholars Program (RSP). The RSP is a yearlong program for early-career faculty with an overall objective to overcome barriers to the academic success, retention, progression, and promotion of groups underrepresented in biomedical and behavioral research. The goal of the RSP is to increase research confidence and productivity, build a supportive research community, and reduce isolation by providing personal and group research enrichment to junior faculty through professional development, mentorship, and networking.

**Methods:**

We adapted evidence-based approaches for our institutional context and vetted the RSP across our campus. The resulting RSP consists of three main elements: (1) five levels of Mosaic Mentorship; (2) group and tailored professional development programming; and (3) scientific and social networking. To determine the potential of the RSP to improve research confidence critical to success, we used a modified shortened version of the Clinical Research Appraisal Inventory (CRAI-12) to assess participants’ confidence in performing a variety of research tasks before and after program participation. We collected information about retention, promotion, and grants submitted and awarded. Additionally, we conducted semi-structured exit interviews with each scholar after program participation to identify programmatic strengths and areas for improvement. Data for Cohorts 1 and 2 (*N* = 12) were analyzed.

**Results:**

Our assessment finds, with one exception, increasing confidence in participants’ research skills across all items, ranging from 0.4 (4.7%) to 2.6 (40.6%). In their exit interviews, the Research Scholars (RS) described their improved productivity and increased sense of belonging and support from others. Research Scholars noted numerous components of the RSP as strengths, including the Mosaic Mentorship model, professional development programming, and opportunities for both informal and formal interactions. Respondents identified time pressure, a lack of feedback, and unclear expectations of the various mentorship roles as areas in which the program can improve.

**Conclusion:**

Preliminary findings indicate that the RSP is successful in building the research confidence of underrepresented and disadvantaged early-career faculty. While this report focuses on the development and protocol of the RSP, additional cohorts and data will provide the evidence base to support dissemination as a national model of research professional development. Such programming is critical to ensure sustainable support structures, institutional networks, infrastructure, and resources that will improve discovery and equity through inclusive excellence.

## Background

A diverse and inclusive scientific workforce enhances research discovery, productivity, and rigor [1]. Most academic research institutions, however, fall short of achieving such diversity, reflecting shortcomings in both hiring and ensuring the research success that enables retention, progression, and promotion [2]. Only 15% of full-time male medical faculty and 19% of full-time female medical faculty identify as members of underrepresented racial and ethnic groups, (defined as “populations that are underrepresented in the medical profession relative to their numbers in the general population”) an increase of just 0.7% since 2010 [3]. Across Research 1 (R1) institutions, an average of 13.1% of faculty overall identified as being from groups underrepresented in academics [4].

Numerous factors account for this lack of representation, including suboptimal recruitment and significant barriers to promotion and retention. While many programs have been developed to improve inclusive recruitment success, fewer programs aim to retain faculty by ensuring scientific and social connections and countering persistent disparities in research success. Underrepresented minority (URM) faculty often feel excluded, invisible, or hyper-visible as representatives of historically underrepresented groups [5]. Inadequate incorporation into scientific and social networks and the lack of a critical mass of diverse faculty may result in isolation and insufficient support [2]. Subpar faculty development programming and substandard mentoring may contribute to challenges in achieving research success [2]. Additionally, faculty may spend extensive time on traditionally undervalued service work, contributing to the “minority tax” [5] that precludes focus on more institutionally prioritized activities, including seeking grant funding. When such grant funding is sought, success rates are lower for URM faculty. For example, the National Institutes of Health (NIH) funding rate for white scientists is nearly 1.7-fold higher than for Black scientists [6].

Despite the continued diversification of graduate students, most academic settings continue to lack appropriate representation of faculty. Nationally, across the biomedical, behavioral and social, and clinical sciences, the URM graduate student population increased from 24.0% in 2013 to 31.1% in 2020 [7]. (These percentages are likely inflated because, in this sample, “Asian” graduate students, who *are not* designated as URM under the current NIH definition, are included in the same demographic category as “Pacific Islander” graduate students, who *are* designated as URM under the current NIH definition.) While our university’s URM graduate student population is far smaller (12.6%), we have witnessed a similar magnitude of growth [8]. Thus, we can conclude that URM faculty underrepresentation does not align with local and national trends of increasing percentages of URM graduate students. Instead, inadequate representation appears to stem from both hiring and retention challenges. From 2013–2020, our university’s retention rate of faculty from all racial/ethnic backgrounds was 60% compared to 25% for URM faculty. To rectify these problems, we examined institutional data, explored the existing workforce literature, and developed a novel program to enhance diversity and inclusiveness in the scientific workforce.

## Methods

### Program development

Faculty climate and exit surveys suggested several factors and shortcomings that contribute to these representational gaps, including inconsistent professional development, lack of rigorous mentorship, inadequate scientific networking, and isolation. As shown in Table 1, multiple barriers to inclusive faculty hiring, retention, and success exist.

**Table 1:** Summary of barriers and facilitators to underrepresented and disadvantaged faculty success^a^

Career stage/ process	Barriers	Facilitators
Applicant identification urand assessment	▪ Outreach limited to Predominantly White Institutions (PWI) and organizations ▪ Inclusivity dependent on values of committee members ▪ Perception of choice between diversity *or* excellence ▪ Lack of inclusive faculty engagement and input	▪ Expansion of outreach efforts ▪ Enhancement of committee membership to be more inclusive ▪ Deployment of data analytics ▪ Inclusion of broad stakeholder input
Recruitment	▪ Inconsistent search processes ▪ Limited diversity on committees ▪ Diversity, Equity, Inclusion, and Accessibility (DEIA) trainings that are too general, lack rigor, and are not assessed ▪ Overly targeted searches that limit faculty pool	▪ Implementation of evidence-based practices in search processes and trainings ▪ Inclusion of community members on committees ▪ Recruitment of a broad scope of candidates with interests that align with institutional strengths
Hiring	▪ Lack of faculty engagement ▪ Insufficient resources provided for startup packages ▪ Unclear expectations for success ▪ Limited expertise on search committee ▪ Only externally funded candidates hired	▪ Equitable and generous start up packages ▪ Identification of factors that contribute to future success ▪ Hiring of candidates with promise, not just funding
Early career	▪ Insufficient research foundation for many new faculty ▪ Dependence on collaborators for success and mentorship ▪ Lack of mentorship ▪ Social and scientific isolation	▪ Opportunities for career planning through coaching and mentorship ▪ Placement of faculty in collaborative research community ▪ Hiring of research clusters ▪ Collaborative opportunities for transdisciplinary research
Career success	▪ Assumption of mastery at mid-career ▪ Few opportunities for leadership ▪ Minority faculty taxed with committee/service work, which is not heavily valued for promotion	▪ Expansive networks for continued and targeted mentorship, sponsorship, and coaching ▪ Leadership opportunities ▪ Space and time to engage in faculty success planning

^a^From data collected through institutional surveys, focus groups, and forums

To address these systemic intra- and inter-institutional issues, in the summer of 2020, the University of Kentucky Office of the Vice President for Research, Office for Diversity and Inclusion in the College of Medicine, Center for Health Equity Transformation, and Office for Faculty Advancement launched the Research Scholars Program (RSP).

#### Programmatic origins and adaptation of the RSP

The RSP combines evidence-based strategies to promote research success for junior faculty, particularly those from groups underrepresented in biomedical and behavioral research (as defined by NIH OD-20–031). The program is based on principles and components common to the Meyerhoff Scholars Program (MSP) [9] and NIH’s Distinguished Scholar’s Program (DSP) [10] and, at our own institution, the Disparities Researchers Equalizing Access for Minorities (DREAM) Scholars Program [11]. Although serving scholars at different career stages, the key components of these programs are consistent with the RSP: multilevel mentoring, cohort-based professional development programming, and scientific and social networking [12–14]. Given the persistence of disparities in URM and other traditionally underrepresented populations in faculty promotion and retention rates at our institution and at the national level, we elected to prioritize research-intensive early-career faculty from underrepresented and disadvantaged groups, with an overall objective of cultivating the research success that enables progression and promotion and with the specific goals of increasing research confidence and productivity, as well as building a supportive research community to reduce isolation.

The components we include in RSP address four shortcomings — 1) inconsistent professional development, 2) lack of rigorous mentorship, 3) inadequate scientific network, and 4) isolation — confirmed through our institutional data as undermining research and career success. To contextually adapt these programs, we developed a prototype of the RSP with the input of a sixteen-faculty-member Faculty Advisory Committee (FAC). The sixteen FAC members represent numerous disciplines, departments, colleges, and backgrounds. Of these FAC members, ten maintained active research programs, six of whom lead large research centers; four of the FAC members lead faculty development programs; and the remaining two FAC members have expertise in DEIA. After these individuals provided extensive review and revisions of the prototype, we vetted the program to receive input from diverse stakeholders during a series of campus events. A special focus of this vetting involved key informant interviews with ten junior faculty, most of whom identified as belonging to groups underrepresented in the research workforce. These key informant interviews were undertaken by two senior faculty members (NS, SW) using a cognitive interviewing approach to solicit input on program components. The process of adaptation took approximately three months, with input from more than sixty faculty members and staff.

#### Eligibility

Eligibility criteria included having a doctoral-level degree, being a full-time assistant or associate professor in any faculty appointment requiring research activity for promotion, with priority given to investigators who had not yet achieved extramural funding as Principal Investigator and being a U.S. citizen or permanent resident. The requirement of U.S. citizenship or permanent residence is consistent with NIH’s eligibility criteria for most career development awards and fellowship programs [15], salient because many junior faculty apply for such opportunities. We prioritized the acceptance of those individuals from underrepresented and disadvantaged backgrounds (according to the NIH’s definition and applicant’s self-report) and required applicants to describe how their participation in the program would contribute to increasing the diversity and inclusive research excellence within their scientific discipline. The response to this prompt played a significant role in determining application acceptance.

#### Application process

Recognizing that the RSP was new and not well known, our campus-wide call for applications described the program, the benefits, and the intended audience: research-intensive (at least 30% of effort) junior faculty, prioritizing those from groups underrepresented and disadvantaged in biomedical and behavioral sciences. We sought assistance in dissemination from university leadership, including research leaders, deans, and department chairs, and encouraged them to actively reach out to eligible faculty members. Submissions included an applicant’s statement of research, a CV, and a description of how their participation would help to cultivate a more inclusive and diverse research workforce. We recruited twice — in 2020 and again in 2022 — for four RSP cohorts. During each of these recruitment periods, all applicants met criteria described above. After assessment by the FAC, all of the applicants were invited to participate, with a new six-person cohort beginning April 1 of each year and lasting twelve months. Given the demand for the program, our desire to include all qualified applicants, and the need to keep the program individualized and intensive, we employed a staggered cohort model. Cohort placement was determined by the applicants themselves and their perceived readiness to benefit from the program. For example, an applicant seeking to submit their first NIH R-series award within months was placed in Cohort 1, while a person who had recently arrived and was still formulating their research ideas was deferred to Cohort 2.

We established four cohorts, with six Research Scholars (RS) in each of the first three, and four in Cohort 4 (which will have two additional members by the time their participation starts in 2024). The small-group composition met our staffing limitations and adhered to recommendations from faculty focus groups, surveys, and exit interviews to build a cohesive and self-reinforcing cohort by limiting the size of the program.

### Program elements

**Fig. 1:**
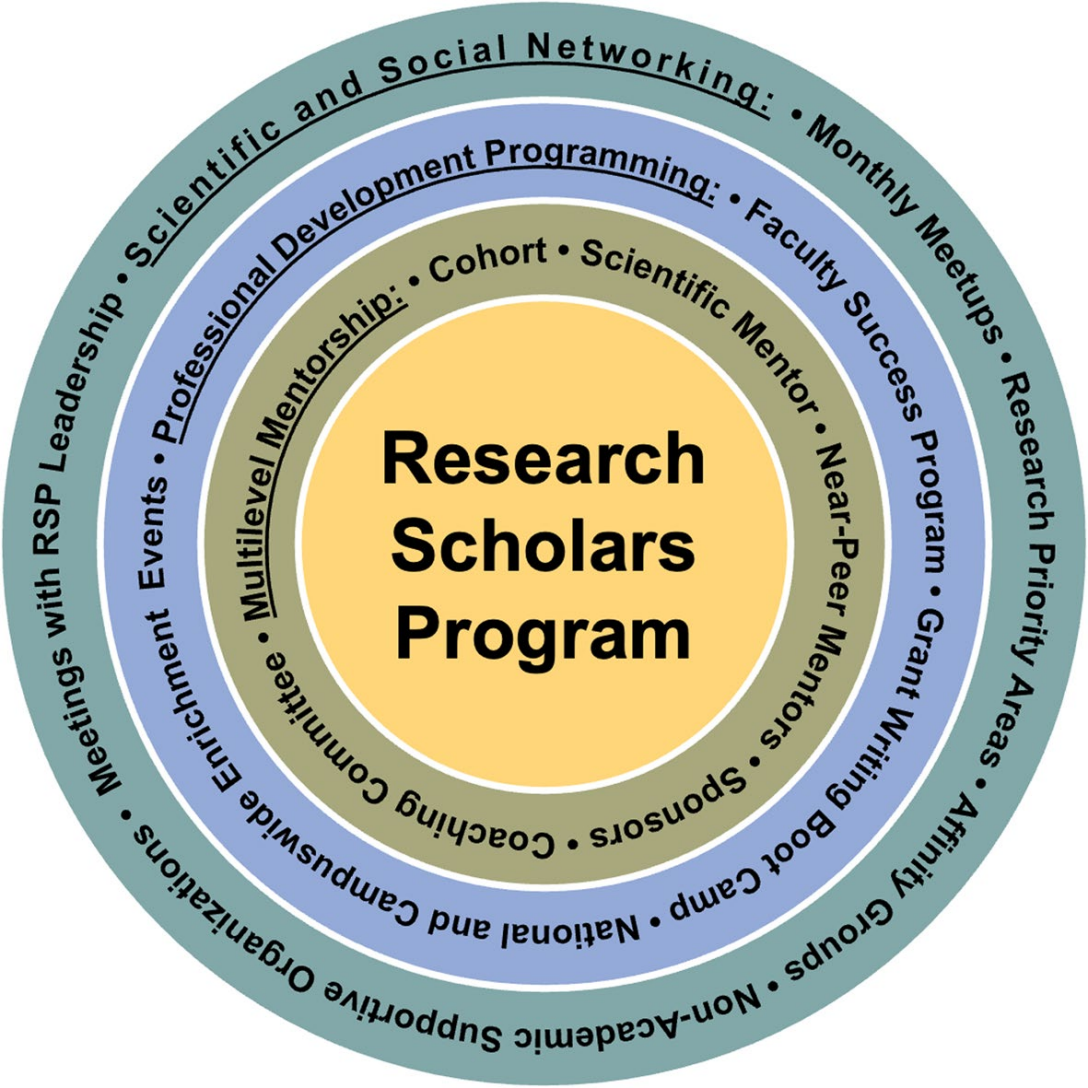
Research Scholars Program (RSP) Components

The RSP consists of three main elements: (1) five levels of Mosaic Mentorship; (2) group and tailored professional development programming; and (3) scientific and social networking. Figure 1 depicts RSP’s components.

#### Mentorship

We draw from the principles of the Mosaic Mentorship model [16], which incorporates five levels of mentorship with multiple individuals. Level one is the cohort itself, a group of highly interactive assistant or associate professors focused primarily on research success. In level two, in consultation with program leadership, RS select a primary scientific mentor who is offered a modest stipend to recognize their commitment to engage in regular (at least monthly) meetings. RS and their scientific mentors develop and sign a Mentoring Compact and Individualized Development Plan. Scientific mentors also are encouraged to complete the eight-week, evidence-based *Entering Mentoring* program [17]. In level three, three to five near-peer assistant or associate professors who have obtained R01-equivalent funding offer quarterly informal sessions on topics selected by the RS, including navigating work-life challenges and achieving research independence. In level four, Sponsors, who are senior scientific leaders of our university’s Research Priority Areas, are asked to advocate and sponsor RS for awards, prizes, and leadership opportunities; enhance their national networks; and provide scientific communities of belonging. Sponsors, representing the areas of cardiovascular disease, diabetes and obesity, cancer, substance use, health equity, and neurosciences, were selected by the RS according to their research interest. For example, several RS with an interest in diet, nutrition, and metabolic disease sought the sponsorship of the Diabetes and Obesity Research Priority Area Leader. Finally, in level five, a Coaching Committee consisting of two senior faculty — selected from the FAC based on career success, familiarity with challenges of underrepresentation in academics, and interpersonal skills — assist RS in navigating the university climate and maintaining a healthy work-life integration. Coaches meet with RS once per semester as a group, or more frequently as needed, to provide RS with advice and tools to advance career development.

#### Professional Development (PD) programming

RS receive rigorous PD programming focused on developing, securing, and executing R01-equivalent research projects. During the first quarter of the RSP, RS are enrolled into the Faculty Success Program (FSP) offered by the National Center for Faculty Development and Diversity. The FSP is a 12-week online program involving weekly calls with a certified coach and three or four peers to share their goals and accomplishments each week. Participants also complete self-directed weekly modules and coach-directed homework while logging into the FSP platform to track their daily progress on research, writing, and personal objectives.

Several grant writing opportunities have been provided to the RS. First, our Proposal Development Office provides group and tailored advice on funding opportunities. These two sessions assist the RS in determining the most appropriate Sponsors (for example, National Science Foundation (NSF) versus NIH or specific institutes within the NIH), mechanisms (Career Development Awards versus R-series grants), and specific opportunities (Requests for Application (RFAs) or Funding Opportunity Announcements (FOAs)) for their proposed projects. Additionally, the first two cohorts joined an institutionally led, 13-week Grant Writing Workshop (GWW) that met weekly for two hours over the final months of their program participation. During the GWW, participants received didactic training on every component of an NIH grant, writing their own section for “homework” that week, and reviewing one another’s documents during the following week. Senior faculty facilitators also provided feedback. While the GWW was extremely thorough, the RS recommended beginning the program during the first few months of program participation, causing us to seek out an alternative program. We commissioned a “Grant Writing Boot Camp” (GWBC) consisting of five intense sessions with similar information for the RS, occurring within the first three months of their participation. Cohort 1 completed only the GWW, while the Cohort 2 completed the GWW and GWBC, Cohort 3 and future cohorts, will participate only in the GWBC since the feedback on the GWBC was so positive.

The FSP and GWBC are supplemented by regular communications from RSP leaders on enrichment events across campus and nationally. These events include a bi-weekly research enrichment program provided by the Office of the Vice President for Research, with topics including Good Clinical Practice, the Secrets of Success for Junior Faculty, and Conducting Humane Research with Animals. Programming through the Center for Clinical and Translational Science (CCTS) provides another enrichment opportunity through the PI 101 course, biostatistical consultation, and the annual CCTS conference. RSP leaders also notify RS of events or organizations that may be of particular interest (for example, the Women in Medicine and Science group or a special workshop).

#### Scientific and social networking

To reduce isolation, provide support for faculty to overcome institutional or structural challenges, and facilitate connections within the research community and institution, the RSP coordinates monthly informal social gatherings. These “monthly meetups” are held at various locations in our community and refreshments are provided by the program. The monthly meetups foster a strong sense of community and allow for RS to share career updates and engage with mentors in an informal setting. The monthly meetups also provide RSP leadership with a venue to continually gather informal feedback on the program and answer pressing questions from the scholars.

To expand their scientific networks, RS also are linked to institutional Research Priority Areas, groups that have been targeted for strategic investment based on existing interdisciplinary strengths, infrastructure, and funding success. Finally, bridging scientific and social networking, RSP leaders conduct a monthly individual meeting with each RS, leaving the agenda to the scholars. Standard topics included hiring personnel for a laboratory, negotiating challenging leaders, and sequencing grant application submissions. The RSP also provides information and referrals on affinity groups and other non-academic supportive organizations.

### RSP costs

Programmatic costs are calculated at $21,600 per faculty, paid by the sponsoring department/college; however, qualified faculty were accepted if the sponsoring department could not afford this cost, and the balance was covered by institutional funds. With the support of university leadership, we selected this budget model so that less well-endowed colleges could offer this program to their faculty members. While this model might lead to some discord (i.e., some departments paying “full price” while others do not), we prefer this model since it equalizes opportunities. Moreover, while all departments have the option of requesting assistance, very few actually request the subsidy. Thus far, of the 18 RS, only four participants (in two colleges/ four departments) have requested subsidized programmatic costs. With documentation of success, we anticipate financial stability through enhanced institutional support and through the receipt of extramural funding. Indeed, with strong backing and financial commitments from university leadership, our team recently has submitted an application to the NIH to support this program. Table 2 provides details on the RSP budget.

**Table 2:** Budget for research scholars program (per participant)

Expense	Estimated expenditures per participant
Faculty Success Program	$4,200
Scientific Mentor payment	$2,000
Near-Peer Mentor payment^a^	$1,000
Coaching^a^	$3,400
Half-time staff^a^	$5,000
Miscellaneous materials and special events^a^	$3,500
Co-directors’ salaries^a^	$2,500
**Total**	**$21,600**

^a^Shared program cost distributed among participants

### Assessment and analysis

Prior to starting the program, we informed RS and their nominators to anticipate allocating 10% of scholars’ effort to participate in the program. No additional departmental or college resources were allocated to protect the scholars’ time. RSP leadership consulted with nominators about how to represent this time allocation, with most considering this effort as unfunded research or professional development.

To determine whether the RSP achieves its stated goals of increasing research confidence and productivity, building a supportive research community, and reducing isolation, leading to overcoming barriers to the academic success, retention, progression, and promotion of underrepresented populations, we implemented a pre- and post-test assessment and exit interviews with the RS and others. Accordingly, we submitted an application to the University of Kentucky Institutional Review Board, approved as protocol #67,403. Participants voluntarily consented prior to completing the pre-test. We acknowledge that standard effectiveness evaluations are essential and we intend to include such evaluations in the future. However, given the program’s promise and the critical need to develop and disseminate information about programs to improve workforce equity, we have opted not to delay and to provide descriptive insights about the RSP.

#### Quantitative assessment

To determine the potential of the RSP to improve research skills and competencies critical to success, we used a modified shortened version of the Clinical Research Appraisal Inventory (CRAI-12) [18] to assess participants’ confidence in performing a variety of research tasks. Pre-test data were collected two weeks prior to program initiation, and post-test data were collected within two weeks after program completion, all via REDCap. RS rated their confidence to successfully perform each item on a scale from 1 (not at all confident) to 10 (extremely confident). Data were analyzed by the RSP evaluation team as anonymized, aggregated datasets, employing descriptive statistics to compare self-rated confidence levels on the successful performance of research tasks.

#### Qualitative assessment

At the conclusion of the program, two RSP FAC members jointly engaged in a 30–60-min virtual, semi-structured interview with each scholar individually. To avoid self-censorship or social desirability bias, no members of the RSP leadership were involved with these interviews. A list of semi-structured interview questions can be seen in Table 3. To ensure anonymity, the sessions were not recorded; instead, one FAC member asked the questions while the other took detailed notes. Given the structured nature of the questions and our goal of program evaluation, the notes were subjected to template coding [19]. Notes were compiled into an aggregated, deidentified dataset based on the question template. Themes were extracted by the RSP leadership team from the template.

**Table 3:** Semi-structured exit interview questions

***Orientation Question***
How would you describe the RSP to people who may want to learn more about it?
***Program Questions***
What were the 1–2 best parts of the RSP? The 1–2 weakest parts of the RSP?
Did the RSP help in your research professional development? If so, how? If not, why not?
As compared to your peers who have not been part of RSP, do you feel that you have received any unique support or experiences? If so, what were these?
Have there been any unique challenges to being a Research Scholar?
***Mentoring***
Can you please share your experiences about the following? (Interviewers are told to gather information related to positive aspects, negative aspects, and potential changes to make.)
- Cohort, Near-Peer Mentors, Scientific Mentors, Sponsors, Coaching Committee
***Formal and Informal Programming***
There are a number of different programmatic elements in the RSP. Can you please share your experiences about the following? Would you share your perspectives on the value of the following program components? (Interviewers are told to gather information related to positive aspects, negative aspects, and potential changes to make.)
- Faculty Success Program, Grant Writing Workshop, Grant Writing Boot Camp, Campuswide and National Enrichment Events, Pitch Session/Lunch and Learn Presentation, Monthly Check-ins with RSP Leadership, Monthly Meetups
***Impact***
Would you recommend RSP to a colleague? What advice would you pass along about the program?
Generally, has the RSP affected your research trajectory? If so, how?
What is the one activity that you felt made the program unique or worthwhile?
If we were cutting one activity or component, what should that be? Why?
What component or activity do you wish we had provided or had provided more of?
Do you feel you are on target for promotion in your college? Please comment
**Wrap-Up Questions**
How might you contribute most effectively to future cohorts of the RSP?
Is there any other feedback you would like to share before we end the interview?

## Results

### Demographics

Table 4 summarizes demographic information for our first three cohorts of RS (*N* = 18). All but one of the RS are assistant professors and are diverse in their professional and personal backgrounds. They hold primary academic appointments in five of six health sciences colleges (Medicine, Nursing, Public Health, Pharmacy, and Health Sciences) as well as the Colleges of Education; Engineering; Arts and Sciences; and Agriculture, Food, and Environment. Twelve (67%) of the RS identify as female and six (33%) as male. Using the standard classification system of the US Office of Management and Budget (OMB) and the NIH [20], one (6%) of the RS identify as Asian, five (28%) as Black or African American, five (28%) as Hispanic or Latino, and eight (44%) as White. Seven RS (39%) are first-generation college graduates, and four (22%) meet two or more of the NIH criteria that define “disadvantaged background” status [21].

**Table 4:** Demographic characteristics of research scholars from cohorts 1, 2, and 3

Demographic Characteristics	Research Scholars (participants) *N* = 18
***Academic Rank***	
Assistant Professor	17 (94%)
Associate Professor	1 (6%)
***Sex (self-identified)***	
Female	12 (67%)
Male	6 (33%)
***Race/ethnicity (self-identified, NIH classifications)***	
Asian	1 (6%)
Black or African American	5 (28%)
Hispanic or Latino	5 (28%)
White	8 (44%)
Prefer not to respond	1 (6%)
***First-Generation College Status***	
First-Generation	7 (39%)
Not First-Generation	10 (56%)
Prefer not to respond	1 (6%)
***Disadvantaged Background Status***	
Meet two or more of NIH criteria	4 (22%)
Do not meet two or more of NIH criteria	13 (72%)
Prefer not to respond	1 (6%)
***College***	
Medicine	5 (28%)
Education	1 (6%)
Public Health	3 (17%)
Nursing	1 (6%)
Health Sciences	2 (11%)
Arts and Sciences	2 (11%)
Engineering	2 (11%)
Agriculture, Food & Environment	1 (6%)
Pharmacy	1 (6%)

Note: Percentages may exceed 100 due to rounding

### Retention rates and career progression

The first two completed cohorts demonstrate early promise with reaching the overall objective of supporting the academic success, retention, progression, and promotion of underrepresented and disadvantaged faculty members. Of the first two cohorts of scholars who have completed RSP, all but one remain at our university (one accepted a faculty position at a prestigious R1 university due to personal circumstances); all (three) who have been eligible for promotion and tenure have succeeded; two have transitioned to tenure track positions; and all eleven have submitted at least two extramural grant applications. Of the approximately 20 grant applications submitted, collectively, three Career Development (K) Awards have been received; four foundation grants have been awarded; and one R01 has been received.

### Quantitative results related to program goals

Table 5 displays mean pretest and posttest RS scores (first two cohorts) on the CRAI-12, including changes over the two time periods (mean scores and percentage change). Pretest and posttest data indicate, with one exception, positive changes in participants’ confidence in their research skills across all items, ranging from 0.4 (4.7%) to 2.6 (40.6%). The most notable improvement related to increased confidence ability to describe ethical concerns with the use of placebos in clinical research (40.6%). Other items with a notable improvement in confidence included: describing major funding agencies’ proposal review and award process (30.8%); identifying faculty collaborators (27.5%); and locating appropriate forms for grant application (18.5%). Those items that demonstrated more modest improvements, and one slight decrease, in confidence included: developing a good analysis strategy (8.1%); asking staff to leave the project team when necessary (6.6%); writing the results section of a research paper (4.7%); and applying the appropriate process for obtaining informed consent (-1.4%).

**Table 5:** Pretest and post-test data from Research Scholars in Cohorts 1 and 2

Item	N	Mean Pretest	Mean Posttest	Change
Design the best research protocol	11	7.3	8.2	+ 0.9 (12.3%)
Develop a good analysis strategy for your study	11	7.4	8	+ 0.6 (8.1%)
Determine an adequate number of subjects for your research project	11	6.9	7.8	+ 0.9 (13.0%)
Write the results section of a research paper	11	8.6	9	+ 0.4 (4.7%)
Write a discussion section for a research paper that articulates the importance of your findings relative to other studies in the field	11	8	8.9	+ 0.9 (11.3%)
Select a suitable topic area for study	10	8	8.8	+ 0.8 (10.0%)
Identify faculty collaborators from within and outside the discipline who can offer guidance to the project	11	6.9	8.8	+ 1.9 (27.5%)
Set expectations and communicate them to project staff	11	7.2	8.2	+ 1.0 (13.9%)
Ask staff to leave the project team when necessary	11	6.1	6.5	+ 0.4 (6.6%)
Locate appropriate forms for grant application	11	6.5	7.7	+ 1.2 (18.5%)
Describe a major funding agency's (e.g. NIH, NSF, or foundation) proposal review and award process	11	6.5	8.5	+ 2.0 (30.8%)
Describe ethical concerns with the use of placebos in clinical research	8	6.4	9	+ 2.6 (40.6%)
Apply the appropriate process for obtaining informed consent from research subjects	10	7.4	7.3	-0.1 (-1.4%)
**Overall**	**-**	**7.2**	**8.2**	** + 1.0 (13.9%)**

Question: Please indicate your ability to successfully perform each task by selecting a single number from one (not at all confident) to ten (extremely confident) that best describes your level of confidence. We would like to know how confident you are that you can successfully perform these tasks today

### Qualitative findings

In their exit interviews, RS noted numerous components of the RSP as strengths. First, RS described how the Mosaic Mentorship model provided them with several supportive layers, each of which conferred a specific benefit. Their own RSP cohort and the Near-Peers gave them reassurance about the challenges faced by early-career faculty. Scientific Mentors, Near-Peers, and Coaches engaged them in candid conversations about navigating academia as a URM or disadvantaged faculty member. The RS indicated that having mentors across faculty ranks proved valuable in cultivating this breadth and depth of support. Second, the Scholars suggested that the PD programming was extremely robust and varied, allowing them to develop a range of critical skill sets, including more consistent writing habits, fundamentals of grant-writing, and skills in “pitching” grant ideas. Third, RS cited the benefits of both formal and informal interactions, which grew their scientific and social networks. More frequent and consistent interactions with Scientific Mentors and Sponsors increased knowledge of funding opportunities and resources while involvement with Near-Peers and their own cohort helped with work-life management. The transdisciplinary nature of the program allowed the scholars to build networks beyond their home discipline and colleges, uncovering potential research collaboration.

RS also described several program limitations. First, several Scholars considered the programming onerous given their numerous faculty responsibilities. Although incoming RS were told that the program would consume at least 10% of their time and that this time would be otherwise be spent on research development, several Scholars felt a great deal of time pressure to balance their many responsibilities. To reduce these time demands, some RS suggested merging meetings, providing more flexible and generous deadlines for task completion, and limiting non-essential PD participation. Second, several of the RS felt that the program provided insufficient feedback and lacked clarity on expectations. These concerns arose in the context of RS’s research proposals and presentations to their Sponsors (“pitch sessions”). Indeed, the impact of the Sponsors constitutes an area for improvement, with RS noting a limited attendance at the pitch sessions and only intermittent nominations for opportunities and awards. Although disappointing, these Sponsors are key leaders at our institution and may lack the time and commitment to fully engage with the RS. Additionally, although the Scholars derived many benefits from their interactions with senior faculty, they recommended providing greater clarity on the expectations between RS, Sponsors, and Scientific Mentors. Accordingly, we have continued to modify the program for each incoming cohort.

## Discussion, limitations and next steps

This article provides an overview of the development process, content, and preliminary findings of a yearlong program designed to promote the research success of junior faculty from groups underrepresented in the health sciences. As described above, the program was developed with the goal of increasing research confidence and productivity, building a supportive research community, and reducing isolation by providing personal and group research enrichment to junior faculty from traditionally underrepresented and disadvantaged groups through professional development, mentorship, and networking. Pre-test and post-test analysis from Cohorts 1 and 2 demonstrated notable improvement in many aspects of research confidence. Some items, including developing a good analysis strategy, asking project staff to leave, and writing a results section, were associated with a more modest improvement across the cohorts or, in the case of applying the appropriate process for obtaining human subjects informed consent, a slight decline. For many of these items, the RS had a relatively high level of confidence at baseline, suggesting a ceiling effect. Qualitative data supported these positive outcomes, as well as provided suggestions for improvement. While limited data and modest sample size preclude us from claiming the RSP’s effectiveness, increased confidence, feelings of belonging and support, nearly complete faculty retention and notable productivity in scholarly products, including grant submissions, suggest promise.

Several observations merit discussion. First, the initial success of the program likely is due to the time-consuming but essential iterative developmental process. During this process, we identified unmet needs and constructed the program to fill those gaps. Although we were responsive to these unmet needs and vetted the program extensively with our FAC, exit interviews and assessments reveal areas for refinement. In response, we provide the grant writing sessions earlier in the program, a stipend to pay an external grant reviewer, more individual (one-on-one) meetings with the Near-Peers and Coaches, and more varying timing for our monthly meetups to allow for those with standing commitments to attend.

Third, although we have presented both a program development process and an outcome (a program design) capable of replication in other environments, it is essential to consider the local context to ensure that resources are optimally used to address limitations in the institutional climate [22]. While the outcomes presented in this descriptive paper are promising and the program potentially exportable to other institutions, we acknowledge that this is a complex, complicated, and relatively resource-intensive program that cannot eliminate centuries of deleterious treatment leading to systematic exclusion from the research workforce. Moreover, because the RSP is only in its third year, our sample size and long term follow up are limited. While we acknowledge limited conclusions can be drawn from a modest sample size, the importance of research workforce inclusion motivates us to share our results without delay.While the RSP appears to be successful, the program alone is not capable of rectifying the underrepresentation of minoritized scientists in academics. Benefitting from the full array of this nation’s talent requires engaging a continuum of inclusivity, from training [23] to hiring, supporting, promoting, and retaining [24]. The RSP was designed to provide junior faculty with mentorship, networking, and professional development opportunities to facilitate their success at one (and only one) necessary point along the continuum. Additionally, while the RSP is resourced appropriately to meet its goals, either future cohorts must remain small, or the program needs more resources to meet the demands of larger cohorts. Moreover, while RSP attempts to continue Scholars’ engagement in aspects of the program, particularly Mosaic Mentorship and scientific and social networking, such a goal requires additional resource allocation. For example, to maintain continuity and support, previous participants continue to receive communications about relevant campuswide and national enrichment opportunities. This continued engagement is not cost prohibitive, but to personalize these enrichment opportunities to RS’s interests would be a challenge as more faculty graduate from the program.

With increasing evidence that the RSP is achieving its goals, we have received support to continue the program and plan to recruit the next cohorts in 2024. While institutional leaders have encouraged us to expand the cohort size, such an expansion depends on our applications for extramural support as well as internal commitments. Thus, while the program is sustainable in its current form, an expansion depends on enhanced institutional commitment.

## Conclusion

In future years and with expanded support from extramural and other sources, the RSP aims to provide more expansive research professional development opportunities. In the future, we will expand our project goals to include our objectives and document outcomes accordingly. These outcomes will include long term follow-up to determine faculty retention and promotion, research success in publications, grants submitted and received, and other indications of scholarly success.

With evidence of success, we aim to export a successful model of inclusive research professional development to other academic settings. Additionally, because our focus group data suggested a critical gap in promotion from associate to full professor, we envision developing sustainable support structures, institutional networks, infrastructure, and resources that will enhance success throughout the career trajectory. With this report, we invite our colleagues to reflect on their own challenges to determine whether the RSP or a similar program might be useful for their institutions. We welcome inquiries and collaboration in multi-institutional analyses of our combined findings. The persistent underrepresentation of the full range of talent in academic health science departments demands nothing less than our collective investment.

## Data Availability

The datasets generated and analyzed during the current study are not publicly available, as they have identifiable information associated with them that would undermine confidentiality. However, the quantitative data can be de-identified and made available from the corresponding author on reasonable request.
